# Clinical Evaluation of the Safety and Efficacy of a 1060-nm Diode Laser for Non-Invasive Fat Reduction of the Abdomen

**DOI:** 10.1093/asj/sjaa418

**Published:** 2021-02-27

**Authors:** Mikaela Kislevitz, Christine Wamsley, Alison Kang, Suzanne Kilmer, John Hoopman, Jennifer Barillas, Jeffrey M Kenkel

**Affiliations:** 1 Department of General Surgery, MedStar Georgetown University Hospital, Washington, DC, USA; 2 Department of Plastic Surgery, UT Southwestern Medical Center, Dallas, TX, USA; 3 Department of Dermatology, University of California, Davis, School of Medicine, Sacramento, CA, USA

## Abstract

**Background:**

Despite the proven efficacy of liposuction, there is a population of patients who prefer non-surgical alternatives. Laser hyperthermia-induced lipolysis has emerged as one non-invasive alternative to liposuction.

**Objectives:**

The authors sought to evaluate the safety and efficacy of a 1060-nm (±10 nm) diode laser for non-invasive fat reduction of the abdomen.

**Methods:**

This single-arm, 2-center study enrolled 30 patients. Patients received a 25-minute 1060-nm diode laser treatment on their abdomen. Ultrasound adipose measurements, body weight, and circumference were taken at baseline and at 6- and 12-week follow-up visits. Blinded evaluators identified “before” and “after” photos of each patient. A patient satisfaction questionnaire was completed by each patient at study exit.

**Results:**

A total 29 patients completed all treatment and follow-up visits. Ultrasound images showed an adipose reduction of 8.55% at 12 weeks post-treatment (*P* < 0.0001). Blinded evaluators correctly identified 67% of the pre- and post-treatment images at site 01 (Sacramento, CA) and 56% at site 02 (Dallas, TX). Satisfaction was high, with 72% of patients reporting being either “satisfied” or “very satisfied” with their results on a 5-point Likert scale. Pain was rated as mild by 62% of patients, moderate by 38%, and severe by none on the Wong-Baker Scale.

**Conclusions:**

These results indicate that a single treatment with a 1060-nm (±10 nm) diode laser, per the treatment protocol, is safe and effective in reducing unwanted fat in the abdomen as objectively measured employing ultrasound. The treatment was well-tolerated among all patients, with minimal discomfort reported and high patient satisfaction.

**Level of Evidence: 4:**

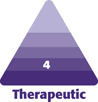

Obtaining an aesthetically pleasing figure has been a driving factor for men and women throughout the years. Both men and women desire the removal of unwanted body fat from the abdomen and contouring of the body. Liposuction remains the gold standard and most utilized intervention for fat removal and improved contour. In 2018, almost 290,000 liposuction procedures were performed by plastic surgeons in the United States, making it the second-most common surgical procedure performed by board-certified plastic surgeons in the United States, ranking only behind breast augmentation.^1^

Despite its documented safety and efficacy, some patients still remain surgically averse or are not candidates for surgery and prefer less invasive or non-invasive procedures with a lower side effect profile and reduced recovery time. The interest in nonsurgical body contouring is growing as is the number of technologies to provide alternative options to surgery, with total expenditures amounting to over $270 million USD in 2018.^1^ Non-surgical fat reduction procedures are the third-most commonly recorded aesthetic non-surgical procedure and include lasers, high-intensity focused ultrasound (HIFU), radiofrequency devices, and cryolipolysis.^1^

Many non-invasive laser technologies have been studied for safety and efficacy in the treatment of unwanted adipose tissue, with the efficacy of laser therapy purported to be related to the wavelength and the energy delivered. Initial laser studies utilized the neodymium-doped yttrium aluminum garnet laser delivering 1064-nm and 1320-nm wavelengths.^2^ The neodymium-doped yttrium aluminum garnet 1064-nm laser energy has low scatter, which allows deeper penetration of the laser energy, creating controlled heating of the tissue in the hypodermis. Diode lasers were first utilized to deliver energy at 900- to 1000-nm wavelengths for long-term hair reduction and the treatment of vascular lesions, for which they were effective. However, they lacked the ability to efficiently and accurately target the chromophores in the focal fat region.^3^ The 1064-nm wavelength has been shown to penetrate to deeper layers of tissue compared with 980 nm.^4^

The main mechanism of action of the 1060-nm laser for lipolysis is heat, which increases localized catabolic rates of the fat cells. This increase in heat breaks down the triglycerides into free fatty acids and glycerol, which are then transported out of the cell via a fatty acid transporter. They then enter the blood and bind to albumin, allowing them to be transported throughout the body and metabolized by cells as needed. Elevating the adipose tissue temperature to 42°C to 47°C initiates an injury and inflammatory response in the tissue within 5 minutes of heat application.^5,6^ Previous investigations have demonstrated that temperatures of 42°C to 47^o^C can be achieved and maintained in subcutaneous adipose tissue employing a 1060-nm laser along with surface cooling while minimally targeting melanin, allowing such a device to be utilized on all Fitzpatrick skin types.^7,8^ The body’s immune response eliminates the cellular debris at the conclusion of the apoptotic process over the course of 6 to 12 weeks.^8,9^ The 1060-nm lasers can target fat in the hypodermis, resulting in a reduction of unwanted subcutaneous fat while sparing overlying dermal tissues. Results can be noted at 6 weeks post-treatment, and the process completes at around 12 weeks post-treatment.^8,9^

This study evaluated the safety and efficacy of a 1060-nm diode laser for non-invasive fat reduction of the abdomen, a common area for patients to seek cosmetic contouring.

## METHODS

### Study Design

The single-arm study consisted of 2 centers (site 01 in Sacramento CA, and site 02 in Dallas, TX), and the study protocols were approved by Western Institutional Review Board, Inc. for site 01 and the Institutional Review Board at the University of Texas Southwestern Medical Center for site 02. The study, completed between February 2019 and June 2020, enrolled 30 patients who received a 25-minute diode treatment to their abdomen employing a 1060-nm laser diode. Baseline photographs, adipose thickness ultrasounds, circumference, and weight were taken and again at the 6- and 12-week post-treatment visits to compare with baseline.

### Patient Recruitment

Male and female volunteers over the age of 18 years with a body mass index score of less than 30, willing to refrain from making major changes in their diet or lifestyle during the study, and interested in non-invasive lipolysis of the abdomen were recruited. Patients were screened for exclusionary criteria, including but not limited to pregnancy in the last 3 months, previous liposuction in the last 12 months, trauma or tattoos in the treatment area, and disorders of the skin. Informed consent was obtained from all patients.

### Investigational Device

**Figure 1.**
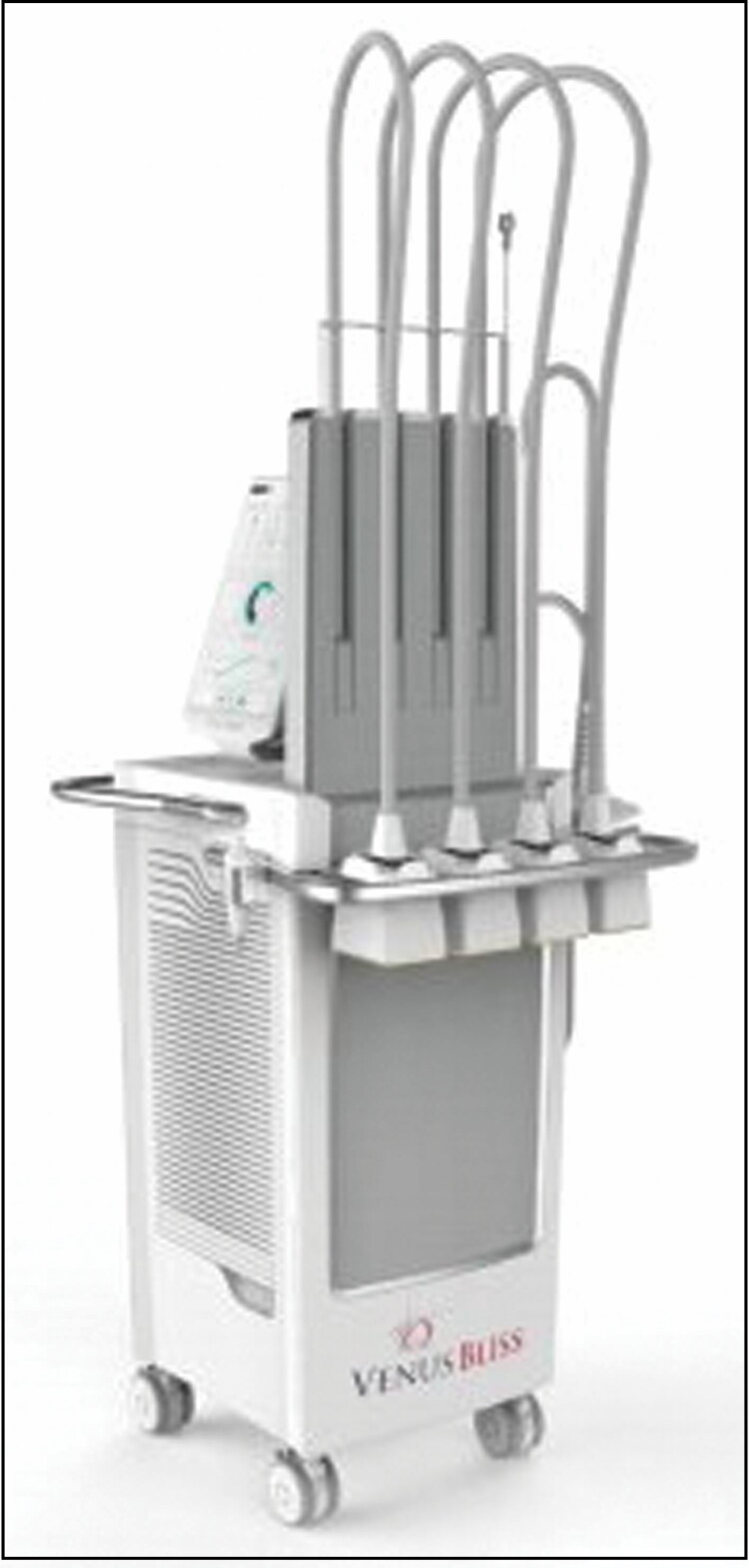
Venus Bliss device. The device consists of four 1060-nm diode laser applicators connected to the main console, which contains a power supply, water-based cooling system, laser driver, and laser controller. The graphical user interface also allows the operator to control device settings on the main console.

The device (Venus Bliss, Toronto, Canada) utilized is comprised of four 1060-nm diode laser applicators connected to the main console, which contains a power supply, a water-based cooling system, laser driver, and laser controller along with a graphical user interface allowing the operator to control the device settings (Figure 1). A belt is utilized to secure the laser applicators on the treatment area, allowing hands-free operation and ensuring the laser always remains in contact with the skin during treatment. Each applicator contains a water-cooled window for contact tissue cooling to prevent the skin from overheating and 4 touch sensors to ensure proper placement and contact during laser emission; the treatment window covers an area of 60 mm × 60 mm. Anesthesia is not required and was not utilized in this study.

**Figure 2.**
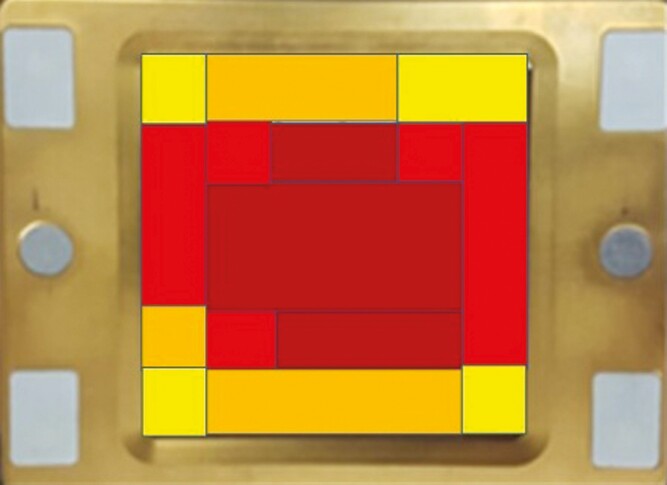
Diode energy distribution over the treatment window. Diagram of energy distribution at the bottom of an applicator. The colored box in the middle represents the treatment window. Colors represent the energy density: 90% to 100% of the energy density is delivered to the areas in dark red zones, 80% to 90% of the energy density is delivered to the areas in lighter red, 70% to 80% of the energy density is delivered to the orange zones, and the yellow zones are 50% to 70%.

The diode energy is distributed over the surface of the applicator’s treatment window, keeping the energy density uniform (Figure 2). This energy dissemination matrix has been created to produce a uniform delivery of energy that facilitates patient comfort and treatment safety.

### Treatment Protocol

Each patient received a single treatment with the investigational device. The location and size of the treatment area were selected based on an assessment of individual patient requirements for treatment. Patients were positioned on their backs with their abdomen area exposed. A belt with frames that hold the diodes in place was affixed to their abdomen with straps and clips. The belt had the capability of holding 4 diodes in place, which was typical for each patient. In smaller patients, only 2 diodes were utilized. Active treatment time was 25 minutes, with each applicator delivering 1.4 watts/cm^2^ and being reduced to as low as 1.1 watts/cm^2^, if necessary, for comfort based on patient feedback.

Baseline photographs, ultrasounds measurements, abdomen circumference, and lifestyle surveys were obtained during the treatment visit prior to the treatment session. Patients presented for follow-up visits at 6 weeks and 12 weeks post-treatment to have photographs taken, weight and circumference recorded, and ultrasound images performed. At the 12-week follow-up, the lifestyle questionnaire was repeated to confirm the patients maintained consistency in their lifestyle for the study duration.

### Clinical Measurements

The study’s endpoints included photographic evaluations by independent blinded evaluators, patient satisfaction surveys, assessment of adverse effects, abdomen circumference measurements, weight, and empirical measurements of adipose thickness at the treated areas utilizing ultrasound.

At baseline and at the final (12-week) follow-up visit, patients completed the modified Simple Lifestyle Indicator Questionnaire to ensure they maintained consistency in their lifestyle for the study duration.^10^ Lifestyle questions assessed diet, alcohol consumption, smoking, stress, and activity levels. Patient satisfaction was evaluated at the 12-week visit employing the Five-Point Likert Patient Satisfaction Scale on a scale from 0 to 4, with 0 being “very unsatisfied” and 4 being “very satisfied.” ^10^ Finally, patients were asked to complete a treatment evaluation questionnaire at the final study visit. Patients were asked whether they noted changes in the treatment area, when post-treatment the changes were noted, the degree of change, any sensations felt during treatment, and how long sensitivity lasted following treatment. All questionnaires were completed on paper when patients presented for their visit.

Adipose layer thickness was measured employing a MicroMaxx (SonoSite Inc., Bothell, WA) with an L38 10-MHz transducer at site 01 and a GE Venue 40 diagnostic ultrasound device (General Electric Healthcare, Wautwatosa, WI) with a 12-MHz transducer at site 02. The measurements were taken in the treatment areas in the same location at each follow-up visit based on photographs with the applicators in place and markings of the applicator locations. The same technician performed each of the ultrasound recordings at each clinical center, with the same minimum required compression force per acceptable standard for ultrasound imaging.^11^ Abdominal circumference was measured utilizing the same tape measure at each visit. Site 01 utilized landmarks on the body and distance from the umbilicus to accurately place the measuring tape around the patient. At site 02, the patient was placed against a measuring tool (Seca 206, Seca, CA) that allowed the investigator to locate the same area for measuring at each visit.

### Blinded Photo Reviews

At baseline and each follow-up visit, photographs were taken of the treatment area from 5 different angles: front facing, right side 90° facing, left side 90° facing, 45° right facing, and 45° left facing. The positions and angles of the photographs were set according to markings of the treated areas to maximize consistency across each visit. Once all follow-up visits had been completed, a set of “before” (baseline) and “after” (12 weeks) photos, each per the above facings, were sent to 3 individual independent evaluators at each clinical center. Site 01 had 1 plastic surgeon and 2 dermatologists, and site 02 had 3 resident plastic surgeons perform the grading. All graders at both sites were blinded to the patients’ identities and the order in which the photos were presented. The graders were asked to identify the sequence of the before and after photos for each patient to the best of their abilities based on visual evaluation. Each grader worked independently and was not aware of the other graders’ scores.

### Safety Evaluations

At regular intervals during the treatment, patients were asked to rate their pain employing the Wong Baker scoring system. The Wong Baker pain assessment scale ranges from 0 (no pain) to 10 (worst pain).^12^ Immediately after treatment, the patients were asked to rate their overall pain perception of the treatment. After removing the belt, the investigator examined the treated areas for hemorrhage, burn, erythema, edema, and purpura using a 5-point scale: 1 = none, 2 = trace, 3 = moderate, 4 = marked, 5 = severe. Patients were asked to report any other self-

observed adverse events at follow-up visits.

### Statistical Analyses

Changes in clinical measurements from baseline to the 6- and 12-week follow-up visits, including weight, body circumference, and adipose thickness, were tested employing Student’s paired *t* test. For the patient satisfaction surveys analyses, Wilcoxon signed rank tests were utilized. Otherwise, descriptive statistics for other objective endpoints were utilized to summarily assess risk and success of the treatment. Unless otherwise indicated, mean ± standard error (SE) are shown.

## RESULTS

### Patient Demographics

A total of 30 patients (15 at each clinical center) were enrolled in the study, of whom 29 completed screening, treatment, and both 6- and 12-week follow-up visits. One patient dropped out during the last follow-up visit prior to completing the ultrasound and body (weight and circumference) measurements, final survey, and lifestyle questionnaire. Of those who completed all visits, there were 26 females (90%) and 3 males (10%). The mean age of patients in the study at the time of enrollment was 45 years, ranging from 26 to 62 years old (Table 1). The Fitzpatrick Skin Type representation was from types I, II, III, IV, and VI, (Table 1) and a body mass index of 25.4 ± 3.1 (mean ± standard deviation [SD]). Race distribution is shown in Table 1. All patients were monitored during the study and completed a lifestyle survey pre-treatment and at the 12-week follow-up visit to ensure their lifestyle was maintained consistently through the duration of the study.

**Table 1.** Patient Age, Fitzpatrick Skin Type, and Race Distributions in Patients Who Completed the Study

Characteristic	Patients (n = 29), No.	Patients, %
Age group, y		
<20	0	0
21-30	1	3
31-40	8	30
41-50	12	40
51-60	6	20
>60	2	7
Fitzpatrick type		
I	1	3
II	13	45
III	9	31
IV	3	10
V	0	0
VI	3	10
Race		
American Indian or Alaska Native	1	3
Native Hawaiian or other Pacific Islander	1	3
Asian	1	3
Caucasian	22	76
Black or African American	2	7
Other	2	7

### Body Measurements

Body weight did not significantly differ between baseline and the 6- and 12-week post-treatment follow-up measurements. The mean weight at baseline was 157.1 ± 5.4 lbs SE, at 6 weeks was 157.3 ± 5.6 lbs SE, and at 12 weeks was 157.1 ± 5.5 lbs SE. Likewise, there was no significant change in body circumference from baseline (36.5 ± 3.5 inches) to either the 6- or 12-week follow-up measurements of 36.7 ± 3.5 inches and 36.5 ± 3.5 inches, respectively.

### Adipose Thickness

**Figure 3.**
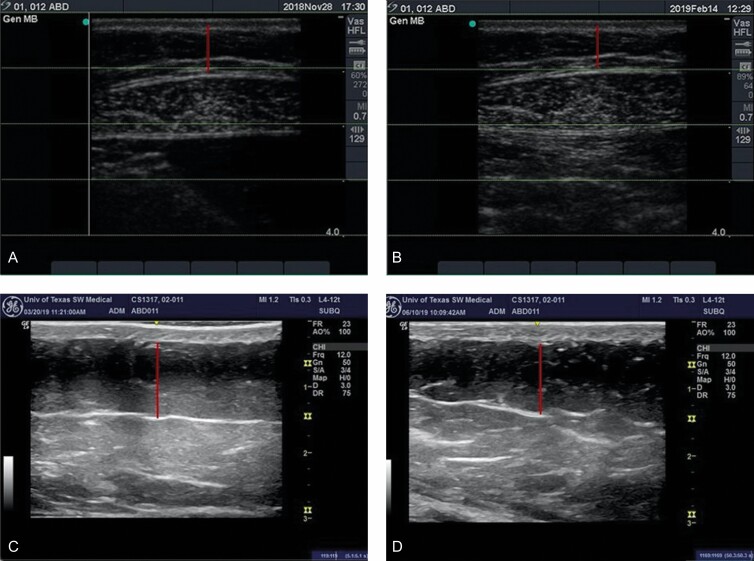
Ultrasound images showing a 12% (from 8.5 to 7.5 mm) decrease between baseline (A) At 12 weeks post-treatment (B) In adipose layer thickness at site 01 and a 9% (from 11.5 to 10.5 mm) decrease in adipose layer thickness between baseline (C) At 12 weeks post-treatment (D) in adipose layer thickness at site 02. Red lines indicate the adipose layer.

**Figure 4.**
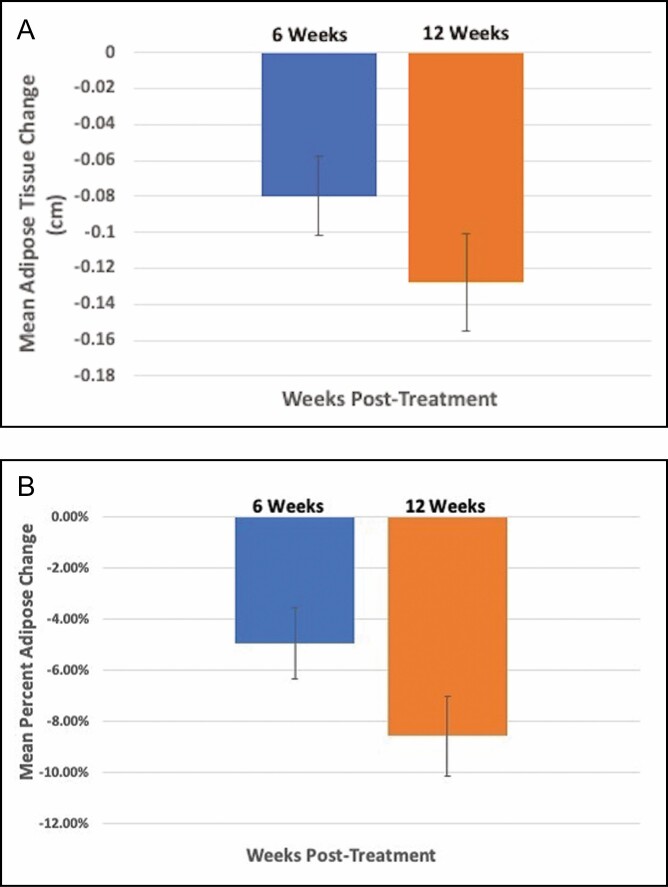
Mean change in the abdominal adipose layer compared with baseline as measured by ultrasound imaging at 6 and 12 weeks post-treatment in centimeters (A) and percent of baseline (B).

Compared with baseline, patients in the study showed a statistically significant reduction in adipose thickness in the treated area in both percent reduction and absolute reduction at 6 weeks (mean reduction 4.92% ± 1.38%, 0.80 mm ± 0.22 mm SE, *P* = 0.001, median 4.2%) and an even greater reduction at 12 weeks after treatment (mean reduction 8.55% ± 1.56%, 1.28 mm ± 0.27 mm SE, *P* < 0.0001, median 9.8%) (Figures 3 and 4; Table 2).

**Table 2.** Adipose Reduction in Millimeters and Percent Improvement as Measured via Ultrasound at 6 and 12 Weeks Post-Treatment Compared With Baseline

	Mean reduction in adipose layer ± SE (mm)	Mean reduction in adipose layer ± SE (% of baseline)
6 Weeks post-treatment	0.80 ± 0.22	4.92 ± 1.38
12 Weeks post-treatment	1.28 ± 0.27	8.55 ± 1.56

mm, millimeters; SE, standard error.

### Photo Reviews

**Figure 5.**
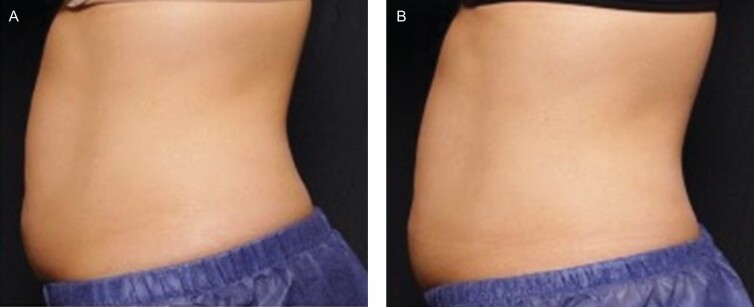
Patient pre-treatment (A) Post-treatment (B) Photographs at 90° from the left side of this 49-year-old female from site 01. Post-treatment photos were taken at the final follow-up visit, 12 weeks after receiving treatment.

**Figure 6.**
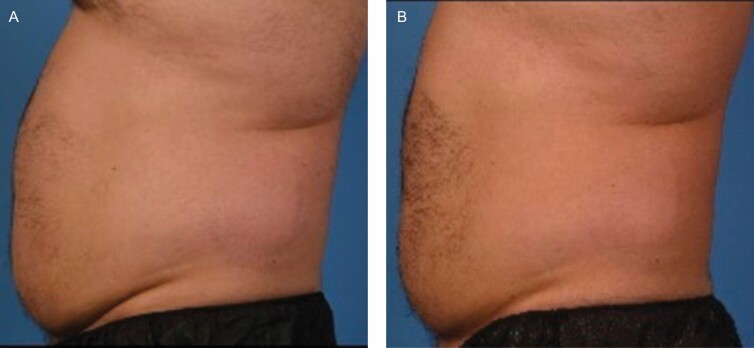
Patient Pre-treatment (A) Post-treatment (B) Photographs at 90° from the left side of this 35-year-old male from site 02. Post-treatment photos were taken at the final follow-up visit, 12 weeks after receiving treatment.

Three blinded evaluators at site 01 correctly identified 67% of the “before” and “after” photos, and 56% of photo sets were correctly identified at site 02. Photo deck examples from both sites are shown in Figures 5, 6. At site 01, there were 15 of 15 patients who completed the before and after photos; however, 1 patient revoked consent to utilize the photos and 1 photo was damaged in the process of taking the photo and was unusable.

### Treatment Pain Assessment

After the treatment was completed, patients were asked to report the level of pain they felt during the treatment employing the Wong-Baker Faces Pain Rating Scale. Patients reported an average (± SD) pain level of 2.6 ± 1.6 both during and immediately post-treatment on an 11-point scale, which translates to “mild pain.” The highest pain rating reported was a 6; however, this was reported in only 2 patients.

### Patient Satisfaction

**Figure 7.**
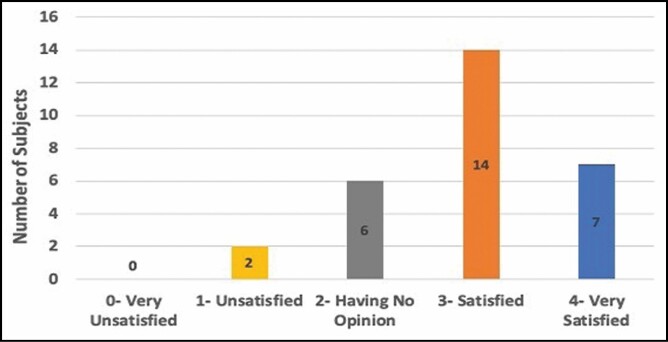
Patient satisfaction response distribution as measured with the 5-point Likert scale 12 weeks after treatment.

All completed patients rated their satisfaction on the last visit of the study prior to exiting the from the study. On a scale of 0 to 4, patients rated their satisfaction at an average of 2.9 ± 0.85 SD, which was a statistically significant satisfaction level and corresponds to “satisfied” on the satisfaction scale. No patients gave a final satisfaction rating of 0 (very unsatisfied), whereas 21 of 29 (72%) of the patients listed their final satisfaction as either 3 “satisfied” or 4 “very satisfied” (Figure 7).

### Patient Evaluation of Treatment and Outcome

**Figure 8.**
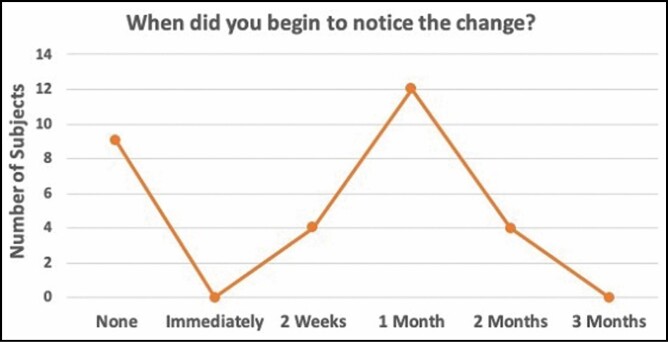
Timeline for patients noticing positive change in the treatment area post-treatment.

**Figure 9.**
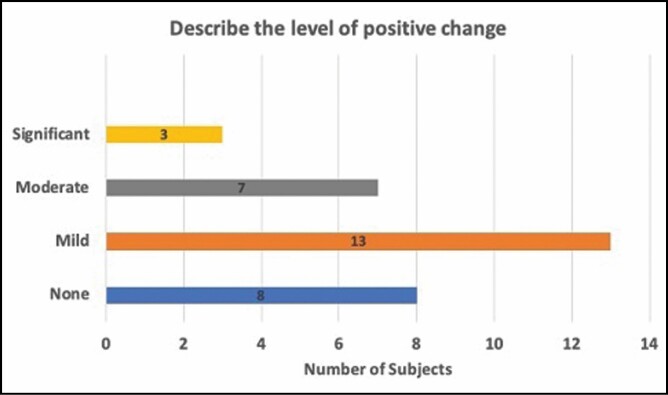
Level of positive change observed by patients 12 weeks post-treatment.

At the end of the study at the final follow-up appointment, patients were given a brief survey to assess their views of the treatment and outcome result. Fourteen percent (14%) of patients began noticing changes as early as 2 weeks after treatment (Figure 8). Forty-one percent, noticed changes 1 month after treatment. Within 2 months of treatment, more than 2 of 3 patients (69%) reported that they saw a change in the treated area of focal fat. Twenty-one (72%) patients stated that they saw a mild to significant level of positive change at the 12-week follow-up visit (Figure 9). The high levels of satisfaction were further supported by 72% of patients stating that they would recommend this treatment to a friend.

**Figure 10.**
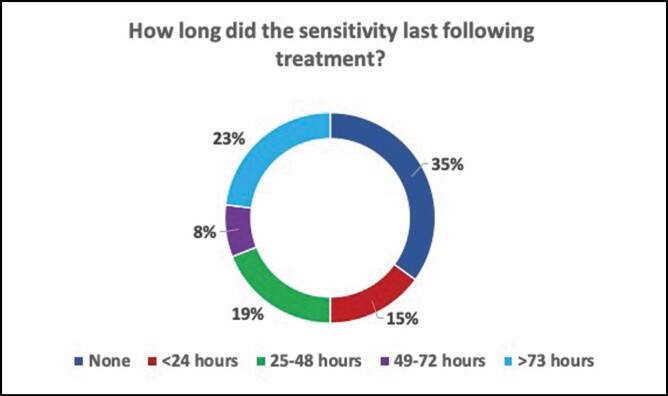
Duration of sensitivity (in hours) post-treatment.

The duration of sensitivity such as heat, stinging, tingling, and burning was limited. Forty-two percent (42%) of patients had sensitivity that lasted 3 days or less, and 35% had no sensitivity. Only 23% experienced sensitivity lasting longer than 3 days (Figure 10). Sensations during treatment varied widely as well, although most reported feeling heat (62%) and fewer patients feeling burning (34%) and tingling (28%).

### Adverse Events

Immediately post-treatment, the investigators visually examined each patient for observed hemorrhage, burn, purpura, erythema, edema, and other visual signs of skin trauma. They rated each event as none = 1, trace = 2, moderate = 3, marked = 4, severe = 5. The investigators reported, as they had expected based on previous experience with laser treatments, erythema in 27 (93%) of the patients; 16 patients had a rating of 2, 10 were rated at a 3, and 1 patient was reported at a 4. Edema was noted in 16 of 29 patients, with 13 patients rated as 2 and 3 patients were rated at 3. Purpura was observed in 1 patient rated as a 2, and possible causes noted were the tightness of the belt and heat response in the tissue.

One anticipated reported side effect was the presentation of nodules in 2 patients at site 02. Site 01 reported no nodules. The nodules were not visible but could be felt upon palpation. Because there was no protrusion or change in skin pigment overlying the nodules, they were not visible in photographs taken at the follow-up visits. The investigator documented 4 nodules of 3 ×3 cm and roughly 2 to 4 fingerbreadths lateral to the umbilicus in 1 patient and 2 nodules bilateral to the umbilicus in the other patient. By study completion, the nodules had either resolved or were reduced in size and were expected to continue diminishing and to resolve over the following months. Nodules are a known occasional side effect of laser lipolysis treatments.^9^ No unanticipated adverse events were reported throughout the course of the study.

## DISCUSSION

Several non-surgical techniques are currently available for patients seeking non-invasive methods of body contouring, including cryolipolysis, HIFU, non-thermal pulsed focused ultrasound, and lasers.^13,14^ All of these technologies trigger an immediate or long-lasting catabolic effect that is either specific or non-specific to the adipocytes, followed by an immune response that removes apoptotic and necrotic cells. Cryolipolysis is based on the premise that adipocytes are more susceptible to cold injury than surrounding water-rich tissue.^14,15^ Macrophages can then remove apoptotic adipocytes, and results can be seen 2 to 4 months post-treatment.^16,17^ HIFU relies on absorbed energy to create molecular vibrations in tissue and generate heat.^18^ The delivery of ultrasonic waves at a frequency of 2 MHz causes targeted coagulative necrosis of adipose tissue. Additionally, microcoagulation is thought to cause collagen remodeling and resultant skin tightening; however, due to high temperatures (up to 65°C during treatment), HIFU requires analgesia and has the potential for nonselective cell necrosis in the target area.^9,13,14,19,20^ Laser technology is a rapidly growing modality for the noninvasive treatment of excess subcutaneous fat.^16^ The results from this 2-center study highlight the safety and efficacy of a single 1060-nm diode laser treatment for non-invasive lipolysis reduction of abdominal fat.

Ultrasound measurements were utilized in this study to determine fat thickness during the study. These measurements are typically considered the gold standard for measuring adipose thickness without the need to anesthetize or perform an invasive procedure on the patients.^11,21^ Patients had a mean 8.55% (1.28 mm) and median 9.8% reduction in the abdominal fat layer seen at 12 weeks post-treatment and up to a 20% reduction seen in some patients; these results are comparable with those in a study with another 1060-nm diode laser.^9^ This was a statistically significant fat reduction after a single 25-minute-long treatment accompanied with no downtime. The treatment was associated with high patient satisfaction, with almost three-quarters of patients reporting they were “satisfied” or “very satisfied” with their results and 72% reporting they would recommend the treatment to a friend. Patient satisfaction is indicative of patients’ perception of a successful treatment outcome.^22^ A key factor for that satisfaction is patient’s recognition of impact. Most patients noticed changes 1 to 2 months post-treatment. Within 2 months of treatment, more than 2 of every 3 patients reported that they saw a change in the treatment area. These findings are aligned with the photographic review by physicians, who were able to identify and distinguish over 61% of all before and after photographs.

Decorato et al^8^ first studied a 1060-nm laser system for abdominal lipolysis and reported the findings of their pilot clinical study in 2017. Hematoxylin and eosin stains of biopsies of the treated abdominal tissue showed evidence of inflammatory changes beginning 5 to 7 days post-treatment. Adipocytes showed signs of injury by day 14. Macrophages surrounded surviving adipocytes at 1 month post-treatment, and by 2 to 3 months post-treatment, foamy macrophages and cystic spaces were clearly visible. By 6 months, fibrosis was apparent and foamy macrophages were decreased in number. Of note, the authors reported no significant changes in serum lipids or liver chemistries as a result of treatment. Additionally, there was a reduction in abdominal fat comparable with that seen after cryolypolysis, as measured by ultrasound and magnetic resonance imaging.^8^ This process of heat-induced adipocyte apoptosis, followed by removal by macrophages, allows a specific reduction of the fat layer. However, there remained the challenge of ensuring patient safety. Some incidences of burns, blisters, and other possible treatment-related ailments have been reported to date with diodes for focal fat treatment.^23^ No incidence of burns or blisters occurred in this study. Although the current study had a limited number of patients, the consistency and intensity of the contact cooling and the homogeneous energy distribution provide uniform energy delivery and impact across the surface of the tissue (Figure 2). This uniform energy distribution, together with the contact cooling, eliminates areas of high peak-power and collective energy impact that are more likely to result in burns and blisters. Most patients experienced transient erythema and edema, which is a common immediate anticipated side effect of diode treatments.^9^ Other side effects, including hemorrhage, were not detected. Two patients did report subcutaneous nodules in the abdominal area within 24 hours after treatment. These nodules are considered typical for laser treatments and have been reported to resolve, on average, within 78 days.^9^ It is likely that these nodules derived from fat lysis as has been observed employing ultrasound. These are possibly fat necrosis aggregates; however, further histological study would need to be completed to confirm this. The treatment area presented with no sharp transitions at the edge areas of the treatment. Sharp demarcation areas around the treatment zone seen with tissue-cooling methods such as cryolipolysis sometimes lead to the appearance of indentations or “shark bite” appearance requiring corrective interventions to smooth out the area.^22^

Patient satisfaction is also derived from comfort during treatment. Cosmetic enhancing solutions often present with moderate discomfort, which can impact the patients’ desire to proceed with certain treatments or to continue with subsequent treatments.^24^ Overall, the treatment in this study was well tolerated, with patients reporting a 2.6 out of 10 on the Wong-Baker Faces Pain Rating Scale. In previous studies employing a different 1064-nm diode device, study patients reported higher pain during treatment with a score of 4 out of 10 on the Wong-Baker Faces Pain Rating Scale.^9^ The difference in comfort levels is likely attributed to the increased cooling and change in energy distribution with the diode utilized in this study (Figure 2).

Patient weight and circumference showed no significant change from baseline to the 12-week follow-up visit, reflecting that patients were consistent with their lifestyle during the study and did not bias the results. This lack of change in weight and circumference was seen despite the 8.55% reduction in fat that was determined utilizing an objective ultrasound measurement of fat thickness. Lack of a significant change in weight is consistent with what has been seen in a comparative study, where a reduction in the abdominal fat layer did not significantly affect the overall body weight or circumference.^9^

Limitations of this study include a smaller sample size lacking racial diversity. Although patient satisfaction was high, with 72% of patients reporting positive change, the reviewers at site 02 were only able to identify the correct pre-treatment and post-treatment images 56% of the time. Furthermore, this study analyzed only a single treatment in 1 area of focal fat. Though the 8.55% reduction in mean adipose thickness is a modest result, this value was statistically significant after a single treatment. Future studies should evaluate the effects of multiple treatments in a single region, which could potentially yield a most robust contour change. Until studies are completed to assess the number of treatments and timing intervals between each session, no definitive conclusions can be drawn about an ideal retreatment regimen. Similarly, larger cohorts could potentially reveal more subtle effects. Some possible changes were potentially masked by this study’s small sample sizes and low statistical power.

## CONCLUSIONS

The results of this study indicate that the 1060-nm diode laser is safe to utilize for the reduction of abdominal adipose tissue and provides statistically significant results even after a single treatment. Changes in adipose thickness were objectively quantified via ultrasound measurements, and patients reported high satisfaction with the treatment and results. Reported side effects were mild and transient, and no major complications were reported over the course of the study. Further studies are needed to assess if 2 to 3 diode treatments will result in greater and more clinically significant abdominal fat reductions.
